# Structural elucidation of a novel arabinogalactan LFP-80-W1 from Lycii fructus with potential immunostimulatory activity

**DOI:** 10.3389/fnut.2022.1067836

**Published:** 2023-01-04

**Authors:** Xiaofei Liang, Mengqiu Liu, Sheng Guo, Fang Zhang, Wanchen Cui, Fei Zeng, Mingming Xu, Dawei Qian, Jinao Duan

**Affiliations:** ^1^Jiangsu Collaborative Innovation Center of Chinese Medicinal Resources Industrialization, Nanjing University of Chinese Medicine, Nanjing, China; ^2^National and Local Collaborative Engineering Center of Chinese Medicinal Resources Industrialization and Formulae Innovative Medicine, Nanjing, China; ^3^Ningxia Innovation Center of Goji R&D, Yinchuan, China

**Keywords:** Lycii fructus, polysaccharide, arabinogalactan, structural elucidation, immunomodulatory activity

## Abstract

Polysaccharides are the most important effective components of Lycii fructus, which has a variety of biological activities and broad application prospects in the fields of medicine and food. In this study, we reported a novel arabinogalactan LFP-80-W1 with potential immunostimulatory activity. LFP-80-W1 was a continuous symmetrical single-peak with an average molecular weight of 4.58 × 10^4^ Da and was mainly composed of arabinose and galactose. Oligosaccharide sequencing analyses and NMR data showed that the LFP-80-W1 domain consists of a repeated 1,6-linked β-Gal*p* main chain with branches arabinoglycan and arabinogalactan at position C-3. Importantly, we found that LFP-80-W1 could activate the MAPK pathway and promote the release of NO, IL-6, and TNF-α cytokines *in vitro*. Therefore, our findings suggest that the homogeneous arabinogalactan from Lycii fructus, can be used as a natural immunomodulator.

## Introduction

The immune system is important for the body to defend against foreign pathogens, and macrophages are the immune effector cells (1). When pathogenic microorganisms invade the host, they synthesize and secrete chemokines and cytokines to enhance the immune defense ability of the body (2). Polysaccharides extracted from traditional medical herbs have attracted extensive attention due to their excellent activation of immune cells and immunomodulatory effects through multiple pathways (3). Notably, they can promote macrophage phagocytosis, induce proinflammatory factors (such as tumor necrosis factor-α, interleukin-1β, and IL-6) and inflammatory mediators (such as nitric oxide) to kill pathogens indirectly, and thus participate in immunomodulatory signaling pathways (4).

Lycii fructus, a famous traditional Chinese medicine tonic for thousands of years, belongs to the dried ripe fruit of *Lycium barbarum* L (5). As a functional food and drug, it is widely used worldwide as a popular nutritional food or healthy dietary supplement, appearing in various forms in fruit juice, wine, tea and various solid foods (6). Lycii fructus polysaccharides (LFPs) have been proven to be the most famous bioactive ingredients of Lycii fructus, which possess antiaging, antidiabetic, antifibrotic, neuroprotective, and immunomodulation properties (7). At the same time, the immunomodulatory effect is primary and is involved in other activities (8). The chemical structure and composition of LFPs have great diversity, and subtle structural changes may affect their immune activity. As such, the immune activity of LFPs is influenced by their monosaccharide composition, molecular weight, and branching degree (9). LBPF4, a polysaccharide-protein complex mainly composed of arabinose and galactose (1.50:2.50), was found to enhance macrophage function by enhancing phagocytosis and activating the NFκB pathway (10). Likewise, a previous study reported that higher molecular weight polysaccharides exhibited better immunomodulatory activity than lower molecular weight polysaccharides extracted from Lycii fructus (11). Additionally, a study has shown that the immunocompetent domains of arabinogalactan LRGP3 seem to be related to the branching degree of the side chain. Compared with the galactan skeleton, the arabinan side chains had stronger macrophage activation activity. In contrast, the inner galactan core significantly increased the complement-fixating activity (12). However, which monosaccharide species or connective structures are closely related to the immune activity is unclear. Therefore, the relationship between the clear structure and immunomodulatory activities of LFPs needs to be further investigated.

In our ongoing search for bioactive polysaccharides, a uniform novel polysaccharide LFP-80-W1 was isolated and purified by column chromatography from Lycii fructus. Different chemical, spectroscopic, and oligosaccharide sequencing characterized its structure. In addition, the immunomodulatory effect of LFP-80-W1 on RAW 264.7 cells and its related target mechanism was also investigated. The results of this study provide useful information for illustrating the structure-function relationship of LFPs and developing immune-enhancing food and drugs from Lycii fructus.

## Materials and methods

### Materials

Dried Lycii fructus was bought from Bairuiyuan Gouqi Co., Ltd. (Yinchuan, China) and identified by Professor Jinao Duan. A voucher specimen (No. LF20210908BRY) was deposited in the Jiangsu Collaborative Innovation Center of Chinese Medicinal Resources Industrialization, Nanjing University of Chinese Medicine, Nanjing, China. DEAE-52 cellulose and Sephacryl S-100 were purchased from Whatman Ltd. (Kent, UK) and GE Healthcare Life Sciences (Piscataway, NJ, USA), respectively. Standard T series Dextrans, monosaccharide standards, sodium cyanoborohydride, and ethyl p-aminobenzoate (ABEE) were purchased from Sigma-Aldrich (St. Louis, USA). Cell counting kit-8 (CCK-8) was purchased from Bioss Biotechnology Co., Ltd. (Beijing, China). Mouse IL-6 and TNF-α ELISA kits were provided by Lianke Biotechnology Co., Ltd. (Hangzhou, China). Primary antibodies against p38, p-p38, ERK, p-ERK, JNK, p-JNK, and β-Tubulin were purchased from Cell Signaling Technology (Boston, USA). The secondary antibody was purchased from Beijing Kangwei Century Biotechnology Co., Ltd. (Beijing, China).

### Extraction, separation, and purification of LFP-80-W1

Air-dried Lycii fructus (10 kg) was defatted with 80% ethanol twice and then extracted twice with reflux water at 100°C. The liquid supernatant was depressurized and concentrated, and the ethanol concentration was adjusted to 30% to precipitate insoluble components such as fiber and pectin. Subsequently, anhydrous ethanol was added to the supernatant to a final concentration of 80% and allowed to precipitate overnight. LFP-80 (163 g) was obtained by freeze-drying and deproteinized by Sevage reagent. Crude LFP-80 (2 g) was redissolved in water and separated on a DEAE-52 cellulose column (4.5 cm × 60 cm) by anion-exchange chromatography. The water-washed fraction was collected and further purified by gel permeation chromatography Sephacryl S-100 column (150 cm × 2.6 cm). The earlier elution part was monitored and collected using the phenol-sulfuric method to obtain the target polysaccharide LFP-80-W1. The protruding peak of the elution curve is shown in Supplementary Figure 1.

### Physicochemical property of polysaccharides

The protein content was determined using the Bradford method with bovine serum albumin as the reference (13). The total sugar content was determined by the phenol sulfuric acid method with glucose as the reference (14).

### Structural characterization of LFP-80-W1

#### Morphological analysis

According to previous literature reports (15, 16), the morphological features of LFP-80-W1 were recorded using a scanning electron microscope (SEM) (Gemini 300, ZEISS, Germany). The acceleration voltage was set at 5.0 kV. LFP-80-W1 solution was dripped on the freshly cleaved mica surface, and scanning probe microscopy images were collected by Atomic force microscopy (AFM) (Dimension Edge, Bruker, Germany).

#### Homogeneity and molecular weight (MW) assays

The molecular weight assay of LFP-80-W1 was determined by high-performance gel permeation chromatography (HPGPC), which was performed on a Waters 2,695 HPLC system (Shimadzu, Kyoto, USA) equipped with a refractive index detector (RID) and two columns (TSK-gel G-3000 and 5000 PW, 8.0 × 300 mm, 6 μM, Showa Denko Co., Tokyo, Japan) connected in series at 35°C. The sample was eluted with 0.01 M PBS buffer at a flow rate of 0.4 mL/min. Furthermore, the Mw was estimated with reference to the calibration curve prepared by standards.

#### Monosaccharide composition assays

The monosaccharides were simultaneously identified and quantitated according to a previously described method (17, 18). Briefly, 5 mg LFP-80-W1 was liberated with 2 M TFA at 110°C for 2 h. The released mixture of monosaccharides was then transformed to corresponding alditol acetate derivatives and separated by a Perkin Elmer Clarus 680 GC system (Perkin Elmer, CA, USA) equipped with an Agilent HP5-MS capillary column (30 m × 0.25 mm × 0.25 μm). The oven temperature was programmed from an initial 100°C for 3 min to 200°C at 20°C/min and held for 2 min, then to 230°C at 5°C/min and held for 2 min, 280°C at 10°C/min for 8 min with helium as the carrier gas.

#### Glycosyl linkage composition analysis

To analyze the composition of glycosyl linkage of LFP-80-W1, methylation analysis was carried out. Briefly, 5 mg vacuum-dried sample of LFP-80-W1 was ultrasonically dissolved in 4 mL DMSO and treated with 500 mg NaOH 0.5 mL of methyl iodide was added and stirred for 2.5 h in the dark. The reaction was inhibited by adding 2 mL of ultrapure water. The methylated polysaccharide was extracted into chloroform and lyophilized to obtain the methylated polysaccharide. The fully methylated products were converted into corresponding partially methylated alditol acetates (PMAAs) derivatives according to Needs’ method (19). The PMAAs were then hydrolyzed with 2 M TFA at 110°C for 2 h, reduced with NaBH_4_, and acetylated with pyridine and acetic anhydride. The acetylated PMAAs were analyzed and identified by the relative retention times and diagnostic GC-MS fragmentation patterns compared with CCRC standard spectral database and previously literature (20, 21).

#### Nuclear magnetic resonance (NMR) analysis

LFP-80-W1 (30 mg) was exchanged and dissolved in 0.55 mL deuterium oxide for NMR analysis. The 1D (^1^H, ^13^C NMR) and 2D spectra (^1^H-^13^C heteronuclear singular quantum correlation HSQC, ^1^H-^13^C heteronuclear multiple bond correlation HMBC, total correlation spectroscopy TOCSY and nuclear overhauser effect spectroscopy NOESY) were achieved on an NMR spectrometer (Bruker AVANCE AV-600, Rheinstetten, Germany) operating at 600 MHz at room temperature.

#### Partial hydrolysis and oligosaccharide sequencing analysis

In this study, ABEE-labeled oligosaccharide fragments were obtained by partial acid hydrolysis and derivatization, and their oligosaccharide sequences were analyzed by UHPLC-QTOF/MS. Briefly, LFP-80-W1 (4 mg) partially was hydrolyzed with 2 mL of 1 mol/L TFA at 70°C for 2 h and the excess acid was removed after hydrolysis. The hydrolyzed sample was mixed with derivatization reagent (0.6 mol/L ABEE, glacial acetic acid and 1.4 mol/L ABEE). The detailed derivatization method was described above (22, 23).

The ABEE-labeled oligosaccharide products were dissolved in 100 μL of 50% methanol, and analyzed on an ACQUITY UHPLC system (Waters, Milford, MA, USA) coupled with a SYNAPT TM Q-TOF detector. Chromatographic separation was performed with an ACQUITY UPLC BEH C_18_ column (2.1 mm × 100 mm, 1.7 μm) with a liner gradient of 0.1% formic acid in water (A) and 0.1% formic acid in acetonitrile (B). The gradient was run for 30 min, 0–2 min, 5% B; 3–5 min, 5–20% B; 5–20 min, 20–30% B; 20–25 min, 20–100% B; 25–28, 100% B; 28–28.1 min, 5% B; 28.1–30 min, 5% B. Several key parameters were set as follows: the ESI ion source works in positive ion mode, the mass scan ranging from *m/z* 100.0000 to 3000.0000, ion source temperature was 120°C, capillary voltage was 3.0 kV, and collision cell collision energy was set to 20 eV.

#### Immunomodulatory activity of LFP-80-W1

##### Cell culture

RAW 264.7 cells were purchased from Shanghai Cell Bank of the Chinese Academy of Sciences (Shanghai, China) and maintained as mycoplasma-free sub-confluent monolayer cultures in RPMI-1640 with 20% FBS at 37°C in a humidified 5% CO_2_ incubator.

##### Cell viability assay

RAW 264.7 cells were seeded in 96-well plates at a density of 1 × 10^5^ cells/mL in triplicate and incubated for 24 h. the cells were then exposed to a series of concentrations of LFP-80-W1 (0, 25, 50, 100, 200, and 400 μg/mL) in serum-free RPMI-1640 for 24 h. cell viability was detected by CCK-8 assay, which shows the mitochondrial activity of living cells by using a microplate spectrophotometer (bio Tek, USA). The absorbance of each well was measured at 450 nm. Cells only suffered medium change were used as vehicle control and normalized to 100% viability.

##### Measurement of NO, IL-6, and TNF-α cytokine production

The RAW 264.7 cells in the logarithmic growth phase were inoculated into a 12-well plate at 2.0 × 10^5^ cells/mL for 24 h. The cells were then treated with lipopolysaccharide (LPS; 1 μg/mL) or LFP-80-W1 at different concentrations (10, 30, and 100 μg/mL) for 24 h or 48 h, respectively. The NO level in the culture supernatant was determined with a commercially available kit based on the Griess reaction. According to the manufacturer’s instructions, the ELISA kit determined the cellular abundances of TNF-α and IL-6.

##### Real-time reverse transcriptase quantitative polymerase chain reaction (RT-qPCR) analysis

Cells were plated in 6-well plates with 5 × 10^5^ cells/mL. Total RNA was extracted using the RNeasy Mini Kit (Tiangen, Beijing, China) in an RNase-free environment. Approximately 1 μg RNA was reverse-transcribed into cDNA using PrimeScript™ RT Master Mix (TaKaRa, Beijing, China). RT-qPCR was performed with the SYBR^®^ qPCR Master Mix (Vazyme, Nanjing, China) (24). Ct values were determined after normalization to housekeeping gene GAPDH mRNA as the invariant control. All the reactions were repeated in three iterations, and relative gene expression multiple was calculated by the 2^–ΔΔ*CT*^ method. The primers (Sangon Biotech, Shanghai, China) used were indicated in Supplementary Table 1.

##### Western blot analysis

Cells were plated in 6-well plates with 5 × 10^5^ cells/mL. The protein samples of the cells were lysed with RIPA buffer (Beyotime, Shanghai, China) containing protease and phosphatase inhibitors. The samples were separated with 20 μg total protein and transferred to cellulose nitrate (NC) membrane at 4°C. The membranes were blocked with TBST containing 5% skim milk for 1 h and probed with primary antibody (1:1,000) against ERK1/2, p-ERK1/2, p38, p-p38, JNK, p-JNK overnight at 4°C, then incubated for an additional 1 h with the secondary antibody (1:5,000). The antibody signal was detected by Bio-Rad chemiluminescence imaging system (California, USA). Software ImageJ was used to quantify and normalize strip intensity.

### Statistical analysis

All data are presented as mean ± SD of a minimum of three independent experiments performed in three biological replicates and analyzed by one-way ANOVA in SPSS 17.0 (SPSS Inc., Chicago, IL, USA) at similar conditions unless otherwise specified. The criterion for statistical significance were *p < 0.05, ^**^p < 0.01 and ^***^p < 0.001.

## Results and discussion

### Purification, homogeneity, and composition of LFP-80-W1

**FIGURE 1:**
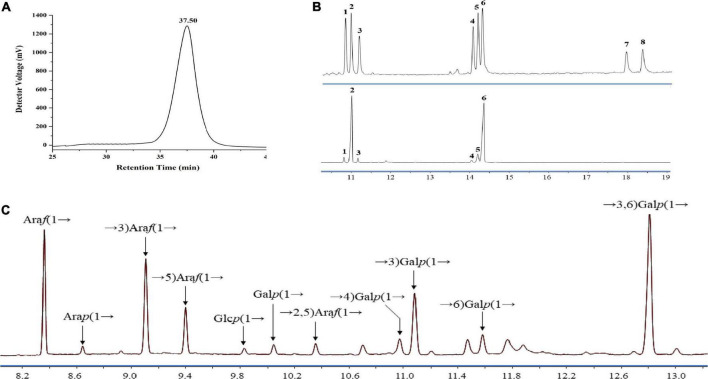
Homogeneity and composition of LFP-80-W1. **(A)** HPGPC spectra; **(B)** mixed standard monosaccharides (upper) and LFP-80-W1 (lower) peak: (1) Rha, (2) Ara, (3) Xyl, (4) Man, (5) Glc, (6) Gal (7) GlcA, and (8) GalA. **(C)** Methylation analysis of LFP-80-W1 and the source data were provided in Supplementary Figure 3 for the identification of each target peak.

Currently, two crude polysaccharides (LFP-30 and LFP-80) were isolated and extracted from the Lycii fructus by fractional alcohol precipitation with a yield of 1.32 and 1.67% based on dry matter. Water soluble polysaccharides LBP-80 were separated by DEAE-Sepharose column chromatography with water elution to obtain the neutral component. The refined LFP-80-W1 (with a recovery rate of 7.50%) was further isolated and purified using Sephacryl S-100. The total polysaccharide and protein content was estimated to be 94.50 and 1.70% by classical phenol-sulfuric acid assay and Bradford method, respectively. LFP-80-W1 showed a continuous symmetrical single peak on HPGPC, and its MW was calculated to be 4.58 × 10^4^ Da by comparing it with the standard dextrans (Figure 1A). LFP-80-W1 was mainly composed of rhamnose, arabinose, xylose, mannose, glucose, and galactose with a molar ratio of 2.97:45.00:3.65:1.06:6.48:40.85, of which arabinose and galactose were the main monosaccharides (Figure 1B). The monosaccharide composition was similar to that of polysaccharides isolated from Lycii fructus as previously reported (25, 26), suggesting that arabinose and galactose may be common monosaccharides commonly contained in Lycii fructus. However, they have structural diversity likely due to different external environments and extraction protocols.

### Methylation analysis of LFP-80-W1

Methylation of polysaccharides is one of the powerful methods to analyze and identify the molecular structure of polysaccharides, which can provide the type and proportion of glycoside bonds. The GC-MS spectra of the PMAAs of LFP-80-W1 are shown in Figure 1C. The results of methylation analysis demonstrated that LFP-80-W1 contained 11 partially methylated glycolol acetate peaks (Supplementary Figure 3 for mass spectra of the targeted peaks). As summarized in Table 1, there are four terminal residues [Ara*f*-(1→, Ara*p*-(1→, Glc*p*-(1→ and Gal*p*-(1→], five linear glycosidic residues [→3)-Ara*f*-(1→, →5)-Ara*f*-(1→, →4)-Gal*p*-(1 →, →3)-Gal*p*-(1→ and →6)-Gal*p*-(1→] and two branching glycosidic residues [→2,5)-Ara*f*-(1→ and →3,6)-Gal*p*-(1→]. The high content of →3,6)-Gal*p*-(1→ indicate LFP-80-W1 may be a highly branched polysaccharide. Ara*f*-(1→ was the primary terminal residue, accounting for 18.82% of the total glycosidic linkages, indicating that the LFP-80-W1 side chains terminated with arabinose residues. In addition, LFP-80-W1 contains numbers of linear polysaccharides, from which it can be inferred that its structure was composed of the main chain and several branches. Some methylation peaks were not accurately identified, probably because of undermethylation or introduced by precolumn derivatization. However, the overall glycosyl linkage composition was consistent with the monosaccharide composition.

**TABLE 1:** Glycosidic linkage composition of LFP-80-W1.

Peak	Glycosyllinkages	RT^a^	PMAA	Fragments(*m/z*)	Mol (%)
1	*Araf*−(1→	8.360	1,4-di-*O*-Ac^b^-2,3,5-tri-*O*-Me^c^ Ara	59,71,87,102,118,129,161	18.82%
2	*Arap*−(1→	8.643	1,5-di-*O*-Ac-2,3,4-tri-*O*-Me Ara	57,71,101,118,131,162	1.30%
3	→3)−*Araf*−(1→	9.106	1,3,4-tri-*O*-Ac-2,5-di-*O*-Me Ara	71,88,101,118,129,161,190	17.14%
4	→5)−*Araf*−(1→	9.398	1,4,5-tri-*O*-Ac-2,3-di-*O*-Me Ara	59,71,87,102,118,129,189	8.08%
5	*Glcp*−(1→	9.830	1,5-di-*O*-Ac-2,3,4,6-tetra-*O*-Me Gal	102,118,129,145,161,205	1.09%
6	*Galp*−(1→	10.047	1,5-di-*O*-Ac-2,3,4,6-tetra-*O*-Me Glc	102,118,129,145,161,205	1.89%
7	→2,5)−*Araf*−(1→	10.356	1,2,4,5-tetra-*O*-Ac-3-*O*-Me Ara	87,129,190	2.08%
8	→4)−*Galp*−(1→	10.973	1,4,5-tri-*O*-Ac-2,3,6-tri-*O*-Me Gal	99,102,113,118,173,233	3.35%
9	→3)−*Galp*−(1→	11.083	1,3,5-tri-*O*-Ac-2,4,6-tri-*O*-Me Gal	101,118,129,161,234	12.13%
10	→6)−*Galp*−(1→	11.583	1,5,6-tri-*O*-Ac-2,3,4-tri-*O*-Me Gal	99,101,118,129,161,173,233	3.94%
11	→3,6)−*Galp*−(1→	12.809	1,3,5,6-tetra-*O*-Ac-2,4-di-*O*-Me Gal	87,101,118,129,160,189,233	30.18%

^a^RT, retention time (min); ^b^Ac, acetyl; ^c^Me, methyl.

### Morphology analysis of LFP-80-W1

**FIGURE 2:**
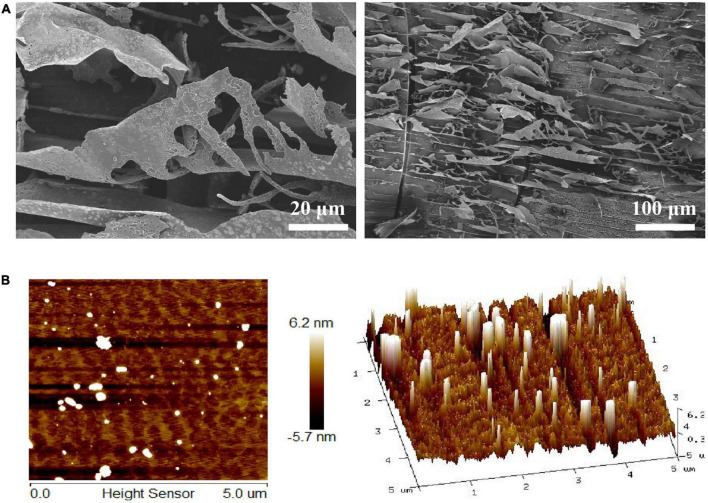
Surface morphology of LFP-80-W1. **(A)** SEM image (500 × and 100 × magnification); **(B)** AFM image and 3D topography.

SEM was the most commonly used technique to investigate the microstructure at a submicron level. SEM obtained the surface morphology of LFP-80-W1, and the results are shown in Figure 2A. LFP-80-W1 showed an irregular thin fragment ship with a loose structure, suggesting the presence of a complex polysaccharide branching structure. AFM analysis is a detection method to directly observe the conformation and three-dimensional surface states of biological macromolecules. The AFM images of LFP-80-W1 are shown in Figure 2B. In the 5 nm field of view, LFP-80-W1 primarily comprised random linear chains and a few spherical aggregations, with conical shapes of varying heights in the 3D image, indicating that intramolecular and intermolecular van der Waals forces and hydrogen bonding resulted in the assembly of LFP-80-W1 molecules into aggregate structures.

### NMR spectroscopy analysis of LFP-80-W1

**FIGURE 3:**
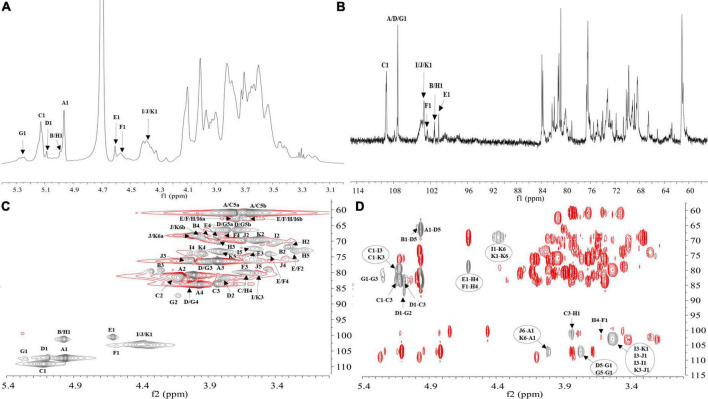
NMR spectra recorded for LFP-80-W1 (600 MHz): **(A)** ^1^H NMR spectrum; anomeric hydrogen signals are annotated. **(B)** ^13^C NMR spectrum; anomeric carbon signals are annotated. **(C)** HSQC spectrum. The most relevant correlations are annotated and marked in gray. A1 represents the cross-peak between H-1 and C-1 of residues A; B1 represents the cross-peak between H-1 and C-1 of residues B and so on. **(D)** HMBC spectrum. The most relevant correlations are annotated and marked in gray. A1-D5 represents the cross-peak between H-1 of residues A and C-5 of residues D; J6-A1 represents the cross-peak between H-6 residues J and C-1 of residues A and so on. (Glycosylated residues A-K are summarized in Table 2).

NMR analysis further characterized the structure of LFP-80-W1. In the ^1^H-NMR spectrum (Figure 3A), 11 anomeric signal peaks located at δ 4.35–5.27 were designated as glycosylated residues A-K, and it can be founded the corresponding 11 anomeric carbon protons ^13^C-NMR (Figure 3B) spectrum.

The high-intensity cross signal at δ 4.96/107.33 in HSQC (Figure 3C) was easily assigned the anomeric signal of residue A [α-L-Ara*f*-(1→] (26). The signal of H-2/C-2 at δ 4.01/80.81 and other signals H-3/C-3, H-4/C-4, and H-5/C-5 could be sequentially assigned and identified with the assistance of the TOCSY spectrum (Supplementary Figure 2A). The complete scalar coupling peak H-1/C-1 of residue C [→3)-α-L-Ara*f*-(1→], observed by the anomeric signal at δ 5.13/109.17, could be determined (27). Similarly, the assignment of residue D [→5)-α-L-Ara*f*-(1→] was suggested by the downfield shifts of δ 66.50 for C-5 in HSQC spectrum (28). The H-1/C-1 of residue G [→2,5)-α-L-Ara*f*-(1→] was assigned by HSQC spectra at δ 5.27/107.42 (29). Chemical shifts of H-3/C-3 and H-6/C-6 confirmed the coexistence of glycosidic linkage residue I [→3)-β-D-Gal*p*-(1→], residue J [→6-β-D-Gal*p*-(1→] and residue K [→3,6-β-D-Gal*p*-(1→], which indicated the cross point between branch and backbone of arabinogalactan (30). According to the cross signal at δ 4.61/100.68 of HSQC spectra, this peak was assigned to the anomeric signal of terminal residues E [β-D-Glc*p*]. The cross signal at δ 4.58/102.48 was identified as terminal residues F [β-D-Gal*p*]. Moreover, the correlation cross-peak of H1/C1 was observed at δ 4.99/101.34, which was related to the residue H [→4)-α-D-Gal*p*-(1→]. However, the H-1/C-1 signal of residue B [α-L-Ara*p*-(1→] was covered by that of →4)-α-D-Galp-(1→.

H-1 and C-3 correlation of →5)-α-L-Ara*f*-(1→ and →3)-α-L-Ara*f*-(1→ cross peak indicated at δ 5.09/83.51 in the HMBC spectrum (Figure 3D). The connective peak of δ 5.12/80.06 illustrating that the anomeric hydrogen of →3)-α-L-Ara*f*-(1→ was glycosidically bonded at the O-3 position of itself, suggested the existence of the sequence of →3)-α-L-Ara*f*-(1→3)-α-L-Ara*f*-(1→. Similarly, the linkages between →5) -α-L-Ara*f*-(1→ to →2)-α-L-Ara*f*-(1,5→, →2,5)-α-L-Ara*f*-(1→ to →3)-α-L-Ara*f*-(1→, and →5)-α-L-Ara*f*-(1→ to →5)-α-L-Ara*f*-(1,2→ were suggested by the presence of δ 5.09/87.10, 5.27/83.51, and 3.79/107.42, respectively. Therefore, these demonstrate the possible existence of the branched fragments →5)-α-L-Ara*f*-(1→5)-α-L-Ara*f*-(1,2→3)-α-L-Ara*f*-(1→ and →5)-α-L-Ara*f*-(1→2)-α-L-Ara*f*-(1,5→. T-α-L-Ara*f* shows rich correlation signals in the HMBC spectrum, the H-1 of α-L-Ara*f* was associated with C-3 and C-5 at δ 4.96/83.51,66.50, which indicated the linkage of α-L-Ara*f*-(1→ to →3)-α-L-Ara*f*-(1→ and →5)-α-L-Ara*f*-(1→ (31). Moreover, the C-1 of α-L-Ara*f*-(1→ was associated with H-6 at δ 3.98,4.01/107.33, suggesting the linkage of α-L-Ara*f*-(1→ to →6)-β-D-Gal*p*-(1→ or α-L-Ara*f*-(1→ to →6)-β-D-Gal*p*-(1,3→. In contrast, T-α-L-Ara*p* has only one correlation signal δ 4.99/66.23, which was the anomeric hydrogen of α-L-Ara*p*-(1→ was linked with C-5 of →5)-α-L-Ara*f*-(1→. In HMBC, H-1 of β-D-Glc*p*-(1→ with C-4 of →4)-α-D-Gal*p*-(1→ at δ 4.61/78.74, showing the linkage of β-D-Glc*p*-(1→ to →4)-α-D-Gal*p*-(1→, which was consistent with the ratio of glycosidic bonds in our methylation analysis. The correlation peak at δ 3.61/102.48 revealed that the H-4 of →4)-α-D-Gal*p*-(1→ linked to the C-1 of T-β-D-Gal*p*. Moreover, the signal of δ 3.83/101.34 indicated that the →4)-α-Gal*p*-(1→ of C-4 position was linked to H-1 of →3)-α-L-Ara*f*-(1→, suggesting the presence of the fragments β-D-Glc*p*-(1→4)-α-D-Gal*p*-(1→3)-α-L-Ara*f*-(1→ and β-D-Gal*p*-(1→4)-α-D-Gal*p*-(1→3)-α-L-Ara*f*-(1→. The observed HMBC correlation of δ 5.13/80.06 suggested that C-1 of residue →3)-α-L-Ara*f*-(1→ was linked to O-3 of →3)-β-D-Gal*p*-(1→ and →3)-β-D-Gal*p*-(1,6→. The correlation of δ 3.53/103.08 indicated C-3 of →3)-β-D-Gal*p*-(1→ was linked to O-1 of residues itself and →3,6)-β-D-Gal*p*-(1→. The branch consisted of →3)-β-D-Gal*p*-(1→ and →6)-β-D-Gal*p*-(1,3→ from the observed correlations of δ 4.38/68.90. Therefore, these results suggest that LFP-80-W1 had the backbone of →3)-α-L-Ara*f*-(1→, →3)-β-D-Gal*p*-(1→, with a branching point at C-3 of →3,6)-β-D-Gal*p*-(1→.

Furthermore, the NOESY spectrum (Supplementary Figure 2B) showed the correlation peaks H-1/H-3 at δ 4.35/3.53 between 1,6-β-D-Gal*p* to 1,3-β-D-Gal*p* and 1,3,6-β-D-Gal*p*, indicating the substitution of the →3,6)-β-D-Gal*p*-(1→ and →3)-β-D-Gal*p*-(1→ residues at the third position by the →6)-β-D-Gal*p*-(1→. The repetitive units of H-1 of →3)-α-L-Ara*f*-(1→ and H-6 of →6)-β-D-Gal*p*-(1,3→ the peak could similarly assign at δ 5.13/4.01.

The C/H chemical shifts of each glycosylated residue were classified in combination with 2D-NMR spectra and are listed in Table 2. The structural similarity of sugar units caused the signal overlapping in the resonance crowded region, and hindered the further structure resolution of LFP-80-W1. According to the existing information, we have assigned the parts without overlap. As the emergence of unambiguous approaches, more refined polysaccharide structures will be revealed in the future.

**TABLE 2:** ^1^H and ^13^C NMR chemical shifts for LFP-80-W1.

Peak	Glycosyl residue	H-1/C-1	H-2/C-2	H-3/C-3	H-4/C-4	H-5/C-5	H-6/C-6
A	α-L-Ara*f*-(1→	4.96/107.33	4.01/80.81	3.82/76.41	3.97/83.74	3.60, 3.72/61.12	
B	α-L-Ara*p*-(1→	4.99/101.34	3.29/71.82	4.24/79.03	3.89/68.29	3.60, 3.72/61.12	
C	→3)-α-L-Ara*f*-(1→	5.13/109.17	4.10/81.12	3.83/83.51	3.63/80.25	3.60, 3.72/61.12	
D	→5)-α-L-Ara*f*-(1→	5.09/107.42	3.79/81.89	3.90/76.61	4.01/83.74	3.66, 3.79/66.50	
E	β-D-Glc*p*-(1→	4.61/100.68	3.25/73.21	3.61/75.90	3.79/68.50	3.44/74.01	3.60, 3.70/62.76
F	β-D-Gal*p*-(1→	4.58/102.48	3.25/73.21	3.60/74.07	3.79/68.50	3.44/74.01	3.60, 3.70/62.76
G	→2,5)-α-L-Ara*f*-(1→	5.27/107.42	4.12/87.10	3.90/76.61	4.01/83.75	3.66, 3.79/66.50	
H	→4)-α-D-Gal*p*-(1 →	4.99/101.34	3.29/71.77	3.82/70.18	3.61/78.74	3.18/72.9	3.60, 3.70/62.76
I	→3)-β-D-Gal*p*-(1→	4.38/103.08	3.41/70.17	3.53/80.06	4.03/73.80	3.55/72.90	3.60, 3.70/62.76
J	→6)-β-D-Gal*p*-(1→	4.35/103.10	3.65/69.78	4.04/76.07	3.32/75.37	3.53/76.31	3.83,3.98/68.90
K	→3,6)-β-D-Gal*p*-(1→	4.38/103.08	3.53/69.71	3.53/80.06	3.94/73.84	3.82/73.21	3.83, 4.01/68.90

### Structure confirmation by oligosaccharide profiles analysis

**FIGURE 4:**
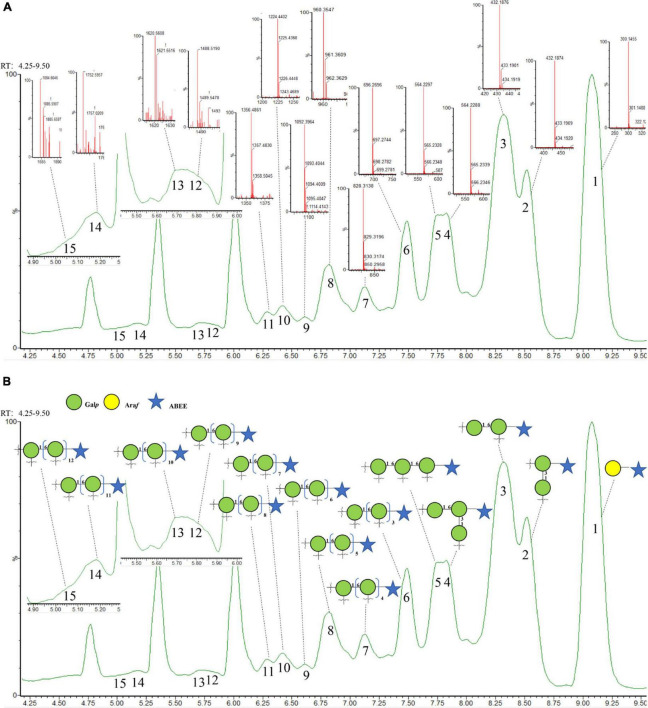
**(A)** Annotated base peak and **(B)** total ion chromatogram of ABEE-labeled oligosaccharides generated from 70^°^C of partial acid hydrolysis.

The dissociation of polysaccharides into constitutive and measurable oligosaccharides has been the critical component for characterizing polysaccharides (32). In order to further confirm the structure of LFP-80-W1, the sequence-specific oligosaccharide fragments obtained were used for polysaccharide identification by tandem mass spectrometry. As shown in Figures 4A, B, fifteen well-separated peaks were shown in the chromatogram. Evidenced by the accurate masses (*m/z*), they represent a sequence of oligosaccharides with increasing degree of polymerization (DP). Peak 1 represented the terminal Ara*f*, and its peak area was consistent with the methylation results, indicating that the terminal junction of LFP-80-W1 was mainly composed of it. Peak 2 and 3 disaccharide isomers were →3)-β-D-Gal*p*-(1→3)-β-D-Gal*p*-(1,6→ and →6)-β-D-Gal*p*-(1→6)-β-D-Gal*p*-(1,3→. The area of peak 3 was higher than that of peak 2, indicating that 1,6-β-D-Gal*p* was the main connection mode of LFP-80-W1. The triosaccharide isomers (peak 4 and 5) were triplicated 1,6-β-D-Gal*p* and 1,3,6-β-D-Galp with galactan at C-3 and C-6 position, respectively. The oligosaccharide spectrum of the remaining peak was linear 1,6-β-D-Gal*p* with 4-13 DP, and each galactose unit was formed by loss of CH_2_O (30 Da) at C-3 position. The detailed information of partial acid hydrolysis and derivatization of LFP-80-W1 has been summarized in Supplementary Table 2.

Compared with enzymatic hydrolysis, partial acid hydrolysis can determine the main chain structure of polysaccharide. Peng et al. obtained arabinan oligomers with 2–7 DP from LRGP3, and ESI-MS analysis showed that the high stability of arabin oligomers toward enzymatic hydrolysis (12). However, in this experiment, there was no arabin oligomers of side chain, only the terminal arabinose was found. Therefore, more information about polysaccharide backbone galactans can be obtained by partial acid hydrolysis.

**FIGURE 5:**
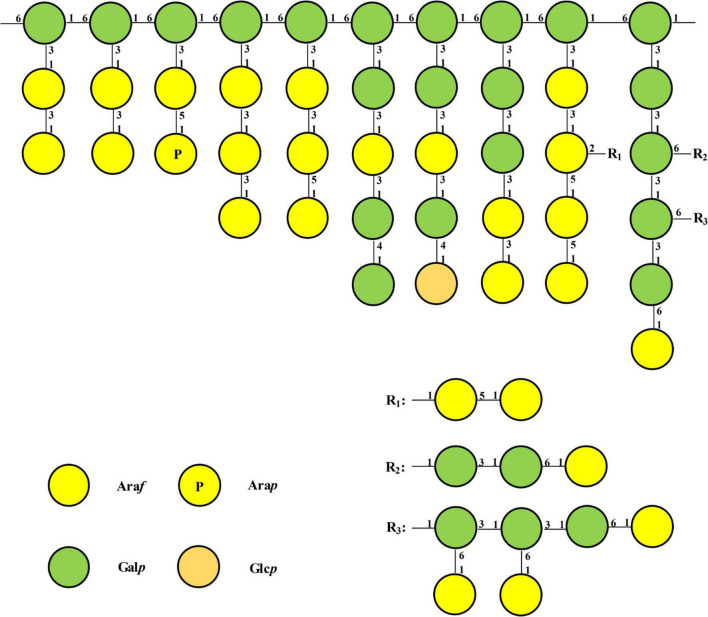
Model structure of possible repeating unit of LFP-80-W1. Only one possible structure was shown, and the connected chains can be arranged in other reasonable orders.

Combining all above results, the probable structure detail of LFP-80-W1 was designed in Figure 5. The structure of the fraction is mainly a carbohydrate chain containing a highly branched arabinogalactan. Its structure was consistent with the backbone of 1,6-substituted linear galactan. Furthermore, the neutral arabinogalactan and arabinan organized the bushy sidechains at C-3 of Gal*p* along the backbone axis.

### Immunomodulatory activity of LFP-80-W1

#### Cell viability of LFP-80-W1 on RAW 264.7 cells

**FIGURE 6:**
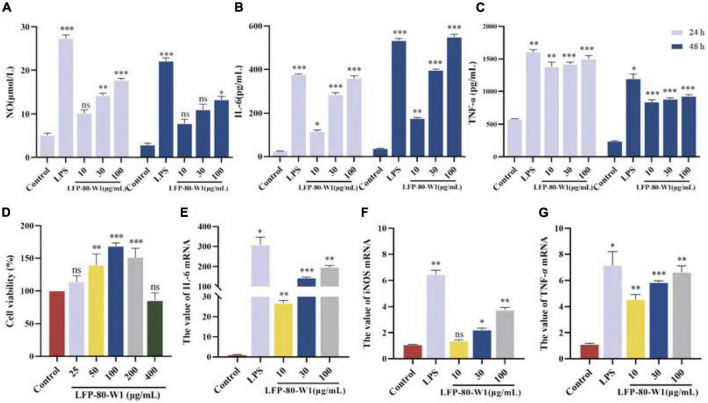
Identification of LFP-80-W1 as a potent immunopotentiator of RAW 264.7 cells. **(A)** The amount of NO produced from the LFP-80-W1 treated RAW 264.7 cells for 24 h or 48 h. Lipopolysaccharide (LPS) 1 μg/mL served as a positive control. **(B)** TNF-α and **(C)** IL-6 from RAW 264.7 cells. **(D)** Effect of LFP-80-W1 on the viability of RAW264.7 cells. The mRNA expression levels of **(E)** iNOS, **(F)** IL-6 and **(G)** TNF-α determined by Real-Time PCR. GAPDH was used as a control. The values are presented as means ± SD (*n* = 3). *p < 0.05, **p < 0.01, and ***p < 0.001 compared with the untreated group.

As shown in Figure 6D, after the treatment with LFP-80-W1 for 24 h, the cell viability rate of RAW 264.7 cells in the concentration range of 25–400 μg/mL was significantly increased in a dose-independent relationship compared with that of the normal control group (*P* < 0.05). When LFP-80-W1 concentration reached 100 μg/mL, the cell viability effect was the most significant, and cell viability began to decline when concentrations began to exceed 100 μg/mL. Thus, polysaccharide concentrations less than 100 μg/mL were used for further investigation.

#### Measurement of NO, IL-6, and TNF-α cytokine production

The secretion of NO, IL-6, and TNF-α in the conditioned medium of RAW 264.7 cells was quantified by ELISA assays to evaluate whether LFP-80-W1 could activate RAW 264.7 cells. Firstly, LFP-80-W1 (10, 30, and 100 μg/mL) could promote NO production in a dose-dependent manner whether treated for 24 h or 48 h (Figure 6A). As shown in Figure 6B, TNF-α were significantly increased in cells produced by different concentrations of LFP-80-W1 and LPS compared with the control group (*P* < 0.05). IL-6 was enhanced when the cells were treated with 30 and 100 μg/mL LFP-80-W1 and LPS at different times (Figure 6C). These results suggest that LFP-80-W1 can activate RAW 264.7 cells to secrete NO, IL-6, and TNF-α *in vitro*, and that the function of LFP-80-W1 was similar to that of LPS.

#### RT-qPCR analysis

To confirm the above observation, an RT-qPCR assay was performed to determine the gene expression of iNOS, IL-6, and TNF-α (Figures 6E–G). After incubation with LFP-80-W1 or LPS for 24 h, the mRNA expression level of iNOS, TNF-α and IL-6 were significantly enhanced and consistent with the result of ELISA assays. The effect of LFP-80-W1 on cytokine release was found to be the most obvious in stimulating IL-6. At the same time, we found that the mRNA expression of iNOS and other cytokines induced by LFP-80-W1 was lower than that of the LPS model control group. Therefore, these findings preliminarily indicated that LFP-80-W1 stimulated the release of NO and inflammatory factors within a specific safe range and did not achieve the effect of causing inflammation.

#### Effects of LFP-80-W1 on the activation of MAPKs signaling pathway

**FIGURE 7:**
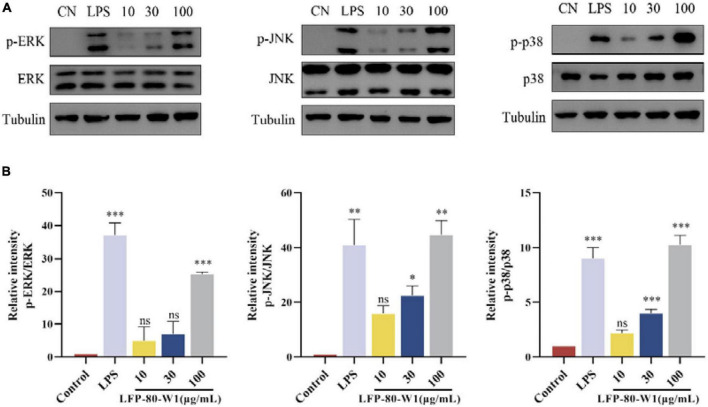
Effects of LFP-80-W1 on the phosphorylation of MAPKs of RAW 264.7 cells. **(A)** Proteins were analyzed by western blot. **(B)** Histograms represent quantification of p38, ERK, and JNK expression levels, respectively. *p < 0.05, **p < 0.01, and ***p < 0.001 compared with the untreated group.

MAPK signaling pathway is a cascade phosphorylation process, which enters the nucleus after activation and regulates transcriptional regulation. The ERK, JNK, and p38 cascade subclasses of MAPK may play an important role in inflammation and apoptosis (33). As shown in Figures 7A, B, the protein activation of MAPK pathway p-p38, p-ERK, and p-JNK levels were promoted when the cells were treated with 10, 30, and 100 μg/mL of LFP-80-W1 or LPS. Moreover, it was clearly identified that LFP-80-W1 at 100 μg/mL caused the highest phosphorylation levels of ERK, JNK, and p38, similar to those in the LPS group. Therefore, these results indicate that LFP-80-W1 could activate the phosphorylation level of key proteins of the MAPK signaling pathway in RAW 264.7 cells.

## Discussion

Arabinogalactans are common components of plant cell walls, characterized by a high proportion of β-D-Gal*p* and α-L-Ara*f* residues (34). Arabinogalactans could be generally divided into linear (1→3)- and (1→6)-β-D-galactan chains based on the differences in backbone linkages, which are substituted at the C-6 and C-3 positions, respectively. Among them, researchers found that the structure contained (1→6)-β-D-galactan as the backbone in the majority, but there were also many reports that the backbone is (1→3)-β-D-galactan. Wu et al. revealed that arabinogalactan was a highly branched polysaccharide from Lycii fructus, and its backbone of (1→6)-β-D-galactan substituted at the C-3 position was linked by α-D-arabinose and β-D-galactan in different ways (25). Yang et al. obtained a Lycii fructus arabinogalactan LBP-3 (Ara*f*: Gal*p* = 1.00:1.56) using DEAE-Crystarose Fast Flow, and its backbone structure was characterized as →1)-β-Galp-(3→ (26). In our previous work, LFPs were water extracted and purified using ion exchange and gel column chromatography to obtain an acidic heteropolysaccharide LFP-1, in which the arabinogalactan domain was highly branched structure with →1)-β-Gal*p*-(3→ as backbone (17).

No matter what the backbone is composed of, the immunological activity of arabinogalactan depends on the type and the degree of branching (35). The multi-branch structure of polysaccharides is a recognition site for the host complement system and an activator of macrophages (36). During our study, LFP-80-W1 had a backbone of 1, 6-Gal*p* and contained approximately 30% of the branches at the O-3 position. The Ara*f* branches mainly include 1,5-linked, 1,3-linked, 1,2,5-linked, and terminal-Ara*f* residues, while the Gal*p* branches mostly contain 1,3-linked, 1,4-linked, and terminal-Gal*p* residues. Furthermore, the bioactivity results have indicated that LFP-80-W1 significantly promoted RAW 264.7 cells proliferation and increased the NO, IL-6, and TNF-α cytokines, suggesting the high branching degree and the length of the branched chain of LFP-80-W1 may be its immunostimulatory active site. Notably, in addition to immune activity, specific structural regions also have a significant impact on anti-aging activity. The anti-aging activity of LFPs decreased after the removal of a large number of arabinose side chains by partial acid hydrolysis, suggesting that branching was an essential part of LBPs to exert anti-aging activity (37).

In fact, the bioactivity of LFPs was closely related to their glycosidic bond. Combined with comparing physicochemical properties and biological activities, we found that LFPs, whose structure contains 1,4-α-D-galacturonic acid and 1,5-α-L-arabinose, play an essential role in immune system regulation (38). Unfortunately, LFP-80-W1 does not contain 1,4-α-D-galacturonic acid. Thus, the contribution of uronic acids to immunostimulatory activity remains elusive. However, LFP-80-W1 includes many 1,5-linked and 1,3-linked α-L-Ara*f* residues, which may explain its favorable immunostimulatory activity. It is worth noting that LFP-80-W1 has a small amount of 1,2,5-linked α-L-Ara*f* residues, which was rare in arabinogalactan. Therefore, arabinose-rich polysaccharides exhibited superiority in immunostimulatory activity.

## Conclusion

The present work revealed the macromolecular structure of novel arabinogalactan LFP-80-W1 purified from Lycii fructus and preliminarily elucidated the immune regulatory mechanism of RAW 264.7 cells. The dominant LFP-80-W1 was a highly branched heterogeneous comprised of a 1,6-linked β-Galp backbone and branched at the O-3 position, with neutral arabinan and arabinogalactan domains. The immunostimulatory effects of exogenous polysaccharide LFP-80-W1 were evaluated at the cellular level. In detail, LFP-80-W1 could dose-dependently increase the intracellular levels of NO, IL-6 and TNF-α and activate the MAPK signaling pathways by upregulating p-ERK, p-JNK, and p-p38. Collectively, these results could contribute to a better understanding of the molecular mechanism of LFP-80-W1 as an effective adjuvant for immunostimulatory activity. Accordingly, Lycii fructus polysaccharides containing LFP-80-W1 may be used to enhance immune function and prevent diseases.

## Data availability statement

The original contributions presented in this study are included in the article/Supplementary material, further inquiries can be directed to the corresponding authors.

## Author contributions

XL: experiment design, performing the experiments, writing—original draft, and formal analysis. ML and WC: performing the experiments and formal analysis. SG: experiment design, formal analysis, writing—review and editing, and funding acquisition. FZh: writing original draft, formal analysis, and writing—review and editing. FZe and MX: writing—original draft and review and editing. DQ: writing—review and editing. JD: formal analysis, writing review—editing, and funding acquisition. All authors contributed to the article and approved the submitted version.

## References

[1] Xie Y Wang L Sun H Wang Y Yang Z Zhang G Polysaccharide from alfalfa activates RAW 264.7 macrophages through MAPK and NF-κB signaling pathways. *Int J Biol Macromol.* (2019) 126:960–8. 10.1016/j.ijbiomac.2018.12.227 30590152

[2] Cao C Zhu B Liu Z Wang X Ai C Gong G An arabinogalactan from *Lycium barbarum* attenuates DSS-induced chronic colitis in C57BL/6J mice associated with the modulation of intestinal barrier function and gut microbiota. *Food Funct.* (2021) 12:9829–43. 10.1039/D1FO01200B 34664587

[3] Yin M Zhang Y Li H. Advances in research on immunoregulation of macrophages by plant polysaccharides. *Front Immunol.* (2019) 10:145. 10.3389/fimmu.2019.00145 30804942 PMC6370632

[4] Sanjeewa K Jayawardena T Kim S Lee H Je J Jee Y *Sargassum horneri* (Turner) inhibit urban particulate matter-induced inflammation in MH-S lung macrophages via blocking TLRs mediated NF-κB and MAPK activation. *J Ethnopharmacol.* (2020) 249:112363. 10.1016/j.jep.2019.112363 31678416

[5] Wu D Guo H Lin S Lam S Zhao L Lin D Review of the structural characterization, quality evaluation, and industrial application of *Lycium barbarum* polysaccharides. *Food Sci Technol.* (2018) 79:171–83. 10.1016/j.tifs.2018.07.016

[6] Dong W Huang K Yan Y Wan P Peng Y Zeng X Long-term consumption of 2-O-β-D-glucopyranosyl-l-ascorbic acid from the fruits of *Lycium barbarum* modulates gut microbiota in C57BL/6 mice. *J Agric Food Chem.* (2020) 68:8863–74. 10.1021/acs.jafc.0c04007 32706586

[7] Wannes W Tounsi M. Phytochemical composition and health properties of *Lycium europaeum* L.: a review. *Acta Ecol Sin.* (2021) 41:390–1. 10.1016/j.chnaes.2020.09.008

[8] Rodrigues C Boldori J Soares M Somacal S Emanuelli T Izaguirry A Goji berry (*Lycium barbarum* L.) juice reduces lifespan and premature aging of *Caenorhabditis elegans*: is it safe to consume it? *Food Res Int.* (2021) 144:110297. 10.1016/j.foodres.2021.110297 34053563

[9] Xiao Z Deng Q Zhou W Zhang Y. Immune activities of polysaccharides isolated from *Lycium barbarum* L. What do we know so far? *Pharmacol Ther.* (2022) 229:107921. 10.1016/j.pharmthera.2021.107921 34174277

[10] Zhang X Li Y Cheng J Liu G Qi C Zhou W Immune activities comparison of polysaccharide and polysaccharide-protein complex from *Lycium barbarum* L. *Int J Biol Macromol.* (2014) 65:441–5. 24530338 10.1016/j.ijbiomac.2014.01.020

[11] Feng L Xiao X Liu J Wang J Zhang N Bing T Immunomodulatory effects of *Lycium barbarum* polysaccharide extract and its uptake behaviors at the cellular level. *Molecules.* (2020) 25:1351. 10.3390/molecules25061351 32188121 PMC7145302

[12] Peng Q Liu H Lei H Wang X. Relationship between structure and immunological activity of an arabinogalactan from *Lycium ruthenicum*. *Food Chem.* (2016) 194:595–600. 10.1016/j.foodchem.2015.08.087 26471597

[13] Bradford MM. A rapid and sensitive method for the quantitation of microgram quantities of protein utilizing the principle of protein-dye binding. *Anal Biochem.* (1976) 72:248–54. 10.1016/0003-2697(76)90527-3 942051

[14] DuBois M Gilles K Hamilton J Rebers P Smith F. Colorimetric method for determination of sugars and related substances. *Anal Chem.* (1956) 28:350–6. 10.1021/ac60111a017

[15] Guo J Song X Wang Y Hua H Ren S Pan Y Isolation, molecular characterization, immunological and anticoagulatant activities of polysaccharides from frankincense and its vinegar processed product. *Food Chem.* (2022) 389:133067. 10.1016/j.foodchem.2022.133067 35490520

[16] Zhang M Qin H An R Zhang W Liu J Yu Q Isolation, purification, structural characterization and antitumor activities of a polysaccharide from *Lilium davidii* var. unicolor Cotton. *J Mol Struct.* (2022) 1261:132941. 10.1016/j.molstruc.2022.132941

[17] Zhang F Zhang X Guo S Cao F Zhang X Wang Y An acidic heteropolysaccharide from Lycii fructus: purification, characterization, neurotrophic and neuroprotective activities *in vitro*. *Carbohydr Polym.* (2020) 249:116894. 10.1016/j.carbpol.2020.116894 32933702

[18] Zhang F Zhang X Liang X Wu K Cao Y Ma T Defensing against oxidative stress in *Caenorhabditis elegans* of a polysaccharide LFP-05S from Lycii fructu. *Carbohydr Polym.* (2022) 289:119433. 10.1016/j.carbpol.2022.119433 35483846

[19] Needs P Selvendran RR. A critical assessment of a one-tube procedure for the linkage analysis of polysaccharides as partially methylated alditol acetates. *Carbohydr Polym.* (1994) 254:229–44. 10.1016/0008-6215(94)84256-6

[20] Liu J Li Y Pu Q Qiu H Di D Cao YL. A polysaccharide from *Lycium barbarum* L.: structure and protective effects against oxidative stress and high-glucose-induced apoptosis in ARPE-19 cells. *Int J Biol Macromol.* (2022) 201:111–20. 10.1016/j.ijbiomac.2021.12.139 34968548

[21] Sims I Carnachan S Bell T Hinkley S. Methylation analysis of polysaccharides: technical advice. *Carbohydr Polym.* (2018) 188:1–7. 10.1016/j.carbpol.2017.12.075 29525144

[22] Wong T Li L Zhang J Zhang Q Zhang X Zhou L Oligosaccharide analysis of the backbone structure of the characteristic polysaccharide of *Dendrobium officinale*. *Food Hydrocoll.* (2022) 134:108038. 10.1016/j.foodhyd.2022.108038

[23] Yan J Shang Z Lv X Du M Ma L Hou G Structure elucidation and antitumor activity of a water soluble polysaccharide from *Hemicentrotus pulcherrimus*. *Carbohydr Polym.* (2022) 292:119718. 10.1016/j.carbpol.2022.119718 35725190

[24] Li Q Chang Y He Z Chen L Zhou X. Immunomodulatory activity of *Ganoderma lucidum* immunomodulatory protein via PI3K/Akt and MAPK signaling pathways in RAW 264.7 cells. *J Cell Physiol.* (2019) 234:23337–48. 10.1002/jcp.28901 31148200

[25] Wu J Chen T Wan F Wang J Li X Li W Structural characterization of a polysaccharide from *Lycium barbarum* and its neuroprotective effect against β-amyloid peptide neurotoxicity. *Int J Biol Macromol.* (2021) 176:352–63. 33549666 10.1016/j.ijbiomac.2021.02.016

[26] Yang Y Chang Y Wu Y Liu H Liu Q Kang Z A homogeneous polysaccharide from *Lycium barbarum*: structural characterizations, anti-obesity effects and impacts on gut microbiota. *Int J Biol Macromol.* (2021) 183:2074–87. 10.1016/j.ijbiomac.2021.05.209 34097961

[27] Ji X Cheng Y Tian J Zhang S Jing Y Shi M. Structural characterization of polysaccharide from jujube (*Ziziphus jujuba* Mill.) fruit. *Chem Biol Technol Agric.* (2021) 8:54. 10.1186/s40538-021-00255-2

[28] Ma W Zhou Y Lou W Wang B Li B Liu X Mechanism regulating the inhibition of lung cancer A549 cell proliferation and structural analysis of the polysaccharide *Lycium barbarum*. *Food Biosci.* (2022) 47:101664. 10.1016/j.fbio.2022.101664

[29] Xia Y Liang J Yang B Wang Q Kuang H. Structural studies of an arabinan from the stems of *Ephedra sinica* by methylation analysis and 1D and 2D NMR spectroscopy. *Carbohydr Polym.* (2015) 121:449–56. 25659720 10.1016/j.carbpol.2014.12.058

[30] Zhou L Liao W Zeng H Yao Y Chen X Ding K. A pectin from fruits of *Lycium barbarum* L. decreases β-amyloid peptide production through modulating APP processing. *Carbohydr Polym.* (2018) 201:65–74. 10.1016/j.carbpol.2018.08.050 30241864

[31] Ji X Guo J Ding D Gao J Hao L Guo X Structural characterization and antioxidant activity of a novel high-molecular-weight polysaccharide from *Ziziphus Jujuba cv. Muzao*. *J Food Meas Charact.* (2022) 16:2191–200. 10.1007/s11694-022-01288-3

[32] Amicucci M Nandita E Galermo A Castillo J Chen S Park D A nonenzymatic method for cleaving polysaccharides to yield oligosaccharides for structural analysis. *Nat Commun.* (2020) 11:3963. 10.1038/s41467-020-17778-1 32770134 PMC7414865

[33] Bi D Yu B Han Q Lu J White W Lai Q Immune activation of RAW 264.7 macrophages by low molecular weight fucoidan extracted from New Zealand *Undaria pinnatifida*. *J Agric Food Chem.* (2018) 66:10721–8. 10.1021/acs.jafc.8b03698 30257559

[34] Li N Yang F Su J Shi S Ordaz-Ortiz J Cheng X Structure characterization of an arabinogalactan from *Cynanchum atratum* and its immune stimulatory activity on RAW 264.7 cells. *Int J Biol Macromol.* (2022) 194:163–71. 34861274 10.1016/j.ijbiomac.2021.11.172

[35] Li N Wang C Georgiev M Bajpai V Tundis R Simal-Gandara J Advances in dietary polysaccharides as anticancer agents: structure-activity relationship. *Trends J Agric Food Chem.* (2021) 111:360–77. 10.1016/j.tifs.2021.03.008

[36] Batbayar S Lee D Kim H. Immunomodulation of fungal β-glucan in host defense signaling by dectin-1. *Biomol Ther.* (2012) 20:433–45. 10.4062/biomolther.2012.20.5.433 24009832 PMC3762275

[37] Huang W Zhao M Wang X Tian Y Wang C Sun J Revisiting the structure of arabinogalactan from *Lycium barbarum* and the impact of its side chain on anti-ageing activity. *Carbohydr Polym.* (2022) 286:119282. 10.1016/j.carbpol.2022.119282 35337529

[38] Xie J Wu D Li W Ning C Tang Y Zhao J Effects of polysaccharides in *Lycium Barbarum* berries from different regions of China on macrophages function and their correlation to the glycosidic linkages. *J Food Sci.* (2017) 82:2411–20. 10.1111/1750-3841.13813 28833151

