# Supplementary material for: Structural elucidation of a novel arabinogalactan LFP-80-W1 from Lycii fructus with potential immunostimulatory activity
Source: Front Nutr. 2023 Jan 4;9:1067836. doi: 10.3389/fnut.2022.1067836 (PMC9846619; doi:10.3389/fnut.2022.1067836)
Supplement: Supplementary file 1 [file Data_Sheet_1.docx]

**Structural elucidation of a novel arabinogalactan LFP-80-W1 from Lycii fructus with potential immunostimulatory activity Xiaofei Liang ^1,2^, Mengqiu Liu ^1,2^, Sheng Guo ^1,2*^, Fang Zhang ^1,2^, Wanchen Cui ^1,2^, Fei Zeng ^1,2^, Mingming Xu ^1,2^, Dawei Qian ^1,2,3^, Jinao Duan ^1,2*^**

*^1^ Jiangsu Collaborative Innovation Center of Chinese Medicinal Resources Industrialization, Nanjing University of Chinese Medicine, Nanjing, China, ^2^* *National and Local Collaborative Engineering Center of Chinese Medicinal Resources Industrialization and Formulae Innovative Medicine, Nanjing, China, ^3^ Ningxia Innovation Center of Goji R & D, Yinchuan, 750002, China.*

** Corresponding author.*

*E-mail address:* guosheng@njucm.edu.cn (S. Guo); *E-mail address:* dja@njucm.edu.cn (J. Duan)

**Supplementary Data**

Table of Contents

**Experimental.** ………………………………………………………………………...3

**Table S1.** The sequences of primers used for Real time PCR analysis…………………4

**Table S2.** Precursors ions and retention time of peaks of LFP-80-W1 based on partial acid hydrolysis and derivatization using UHPLC-QTOF/MS.…………………………4

**Figure S1.** Separation and elution curve of LFP-80-W1………………………………4

**Figure S2.** NMR spectra recorded for LFP-80-W1: (A) TOCSY spectrum; (B) NOESY spectrum ……………………………………………………………………………...5

**Figure S3.** Total ion chromatogram (A) and corresponding mass spectra (B-L) of PMAAs for LFP-80-W1……………………………………………………………6-17

**Figure S4.** IR spectra of LFP-80-W1……………………………………………….18

**Figure S5.** The full HSQC (A), HMBC (B), TOCSY (C) and NOESY (D) spectrum…………………………………………………………………………….19

**Experimental:
The detailed description of Fourier-transform infrared (FT-IR) spectrometric analysis**The IR spectrum were recorded using an FT-IR spectrometer (Thermo Co., Madison, WI, USA) to analyze the organic functional groups in the range of 4000 to 500 cm^-1^.

**Table S1. The sequences of primers used for Real time PCR analysis**

| Gene | Upstream primer sequence | Downstream primer sequence |
| --- | --- | --- |
| GAPDH | GGTTGTCTCCTGCGACTTCA | TGGTCCAGGGTTTCTTACTCC |
| iNOS | ATCTTGGAGCGAGTTGTGGATTGTC | TAGGTGAGGGCTTGGCTGAGTG |
| IL-6 | CTCCCAACAGACCTGTCTATAC | CCATTGCACAACTCTTTTCTCA |
| TNF-α | ATGTCTCAGCCTCTTCTCATTC | GCTTGTCACTCGAATTTTGAGA |

**Table S2.** **Precursors ions and retention time of peaks of LFP-80-W1 based on partial acid hydrolysis and derivatization using UHPLC-QTOF/MS.**

| **No.** | **t_R_ (min)** | **[M+H] ^+^** | **Formula** | **DP** |
| --- | --- | --- | --- | --- |
| 1 | 9.079 | 300.1455 | C_14_H_22_NO_6_ | 1 |
| 2 | 8.521 | 432.1874 | C_19_H_30_NO_10_ | 2 |
| 3 | 8.324 | 432.1876 | C_19_H_30_NO_10_ | 2 |
| 4 | 7.828 | 564.2288 | C_24_H_38_NO_14_ | 3 |
| 5 | 7.747 | 564.2297 | C_24_H_38_NO_14_ | 3 |
| 6 | 7.487 | 696.2696 | C_29_H_46_NO_18_ | 4 |
| 7 | 7.136 | 828.3138 | C_34_H_54_NO_22_ | 5 |
| 8 | 6.816 | 960.3547 | C_39_H_62_NO_26_ | 6 |
| 9 | 6.611 | 1092.3964 | C_44_H_70_NO_30_ | 7 |
| 10 | 6.425 | 1224.4402 | C_49_H_78_NO_34_ | 8 |
| 11 | 6.281 | 1356.4861 | C_54_H_86_NO_38_ | 9 |
| 12 | 5.835 | 1488.5190 | C_59_H_94_NO_42_ | 10 |
| 13 | 5.701 | 1620.5608 | C_64_H_102_NO_46_ | 11 |
| 14 | 5.174 | 1752.5957 | C_69_H_110_NO_50_ | 12 |
| 15 | 5.061 | 1884.6046 | C_74_H_118_NO_54_ | 13 |


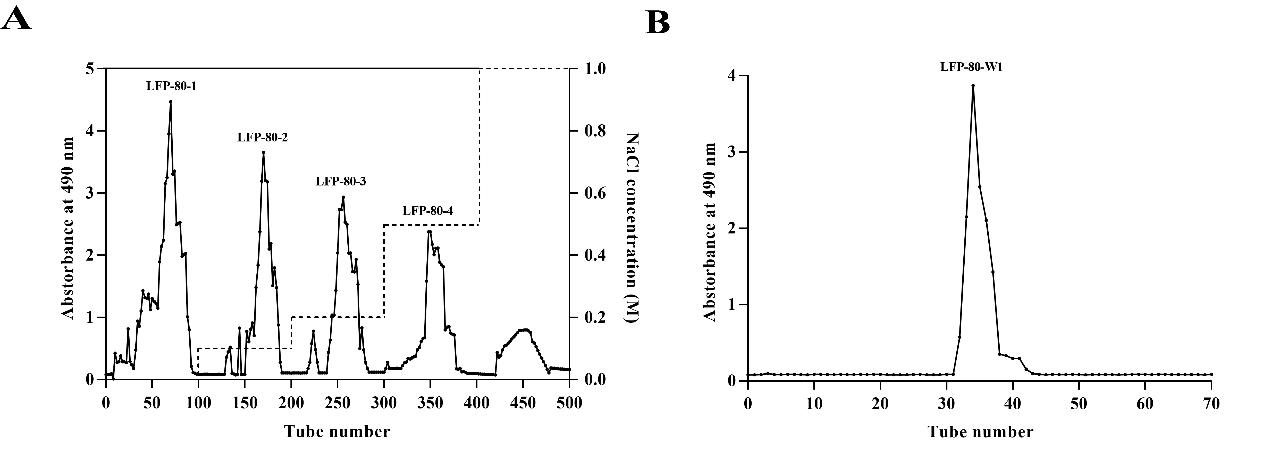


**Figure S1.** Isolation and purification of crude polysaccharides from Lycii fructus. (A) The elution profile of LFP-80 fractions on the DEAE-52 cellulose column (150 × 2.6 cm). (B) The elution curve of LFP-80-W1 component on the Sephacryl S-100 column (150 × 2.6 cm).


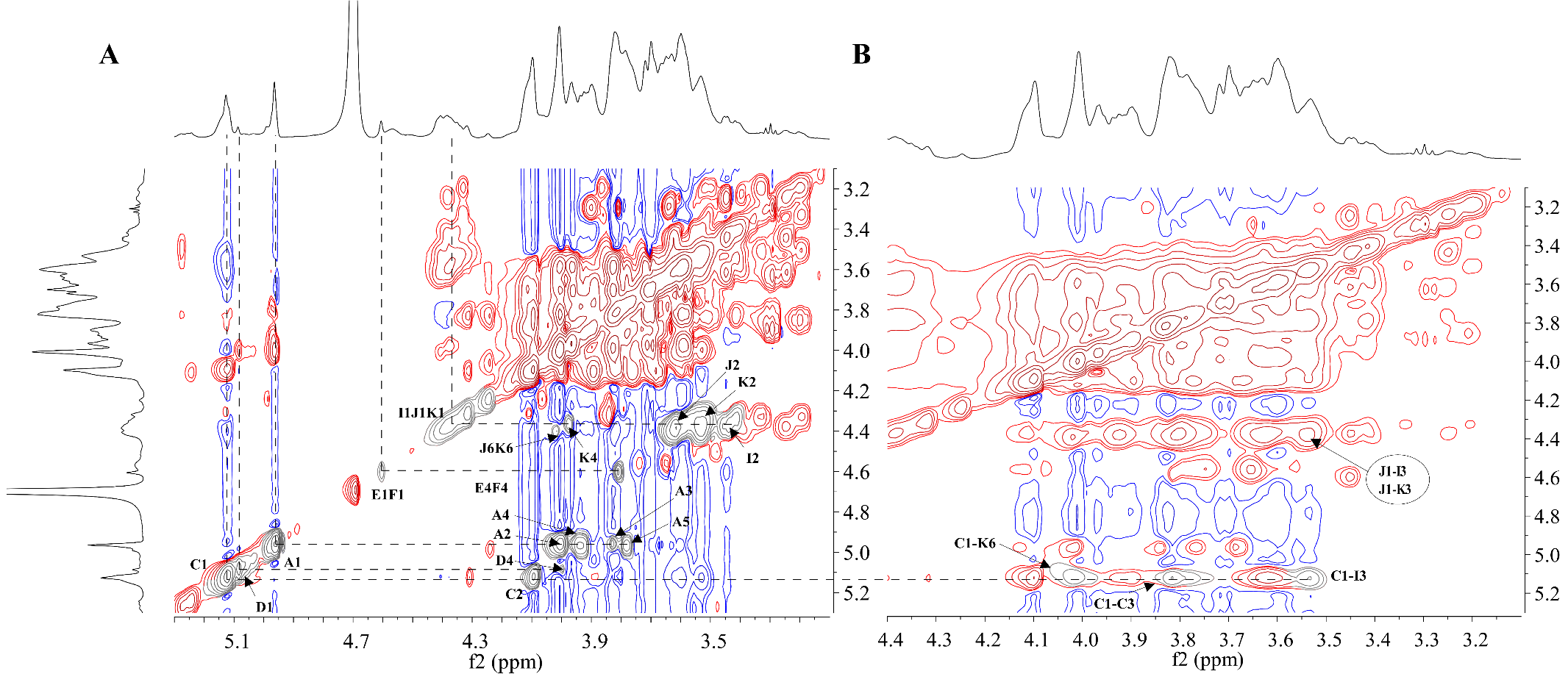


**Figure S2.** NMR spectra recorded for LFP-80-W1: (A) TOCSY spectrum; The most relevant correlations are annotated and marked in grey. A1-A5 represents the cross-peak between H-1 to H-5 of residues A and so on. (B) NOESY spectrum; The most relevant correlations are annotated and marked in grey. C1-C3 represents the cross-peak between H-1 and H-3 of residues C; C1-K6 represents the cross-peak between H-1 of residues C and H-6 of residues K and so on. (Glycosylated residues A-K are summarized in **Table 2**).


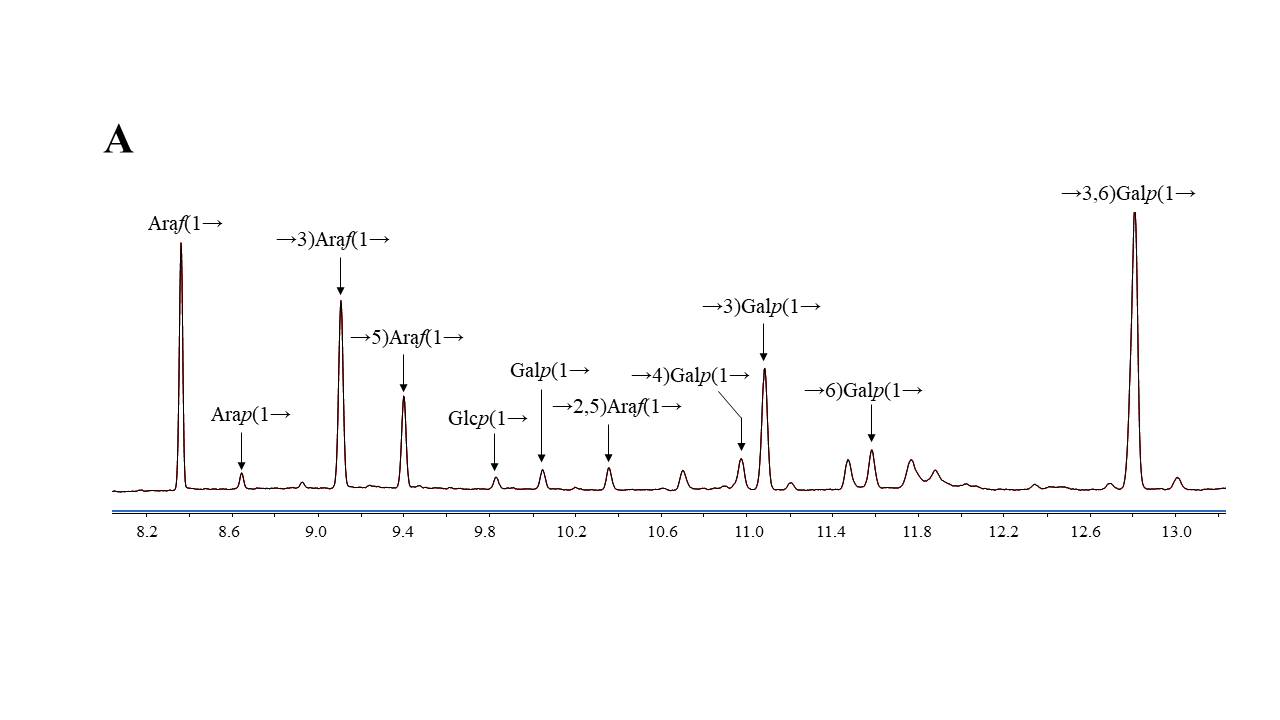


**Figure S3.** Total ion chromatogram (A) and corresponding mass spectra(B-L) of PMAAs for LFP-80-W1


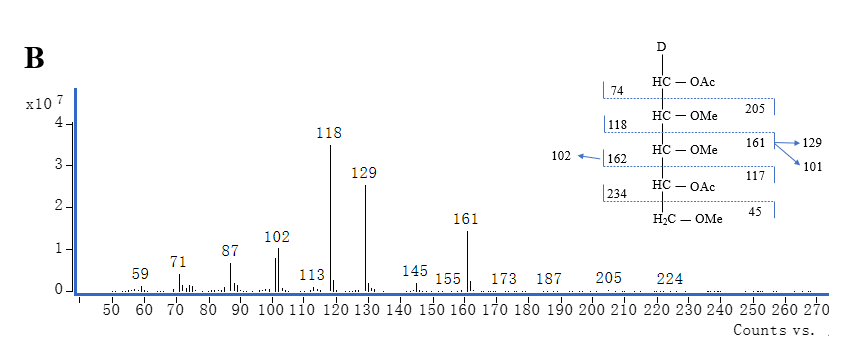


**Figure S3.** Mass spectra of peak at 8.360 min; PMAA: 1,4-di-*O*-acetyl-2,3,5-tri-*O*-methyl arabinitoltype of linkage: Ara*f*-(1→


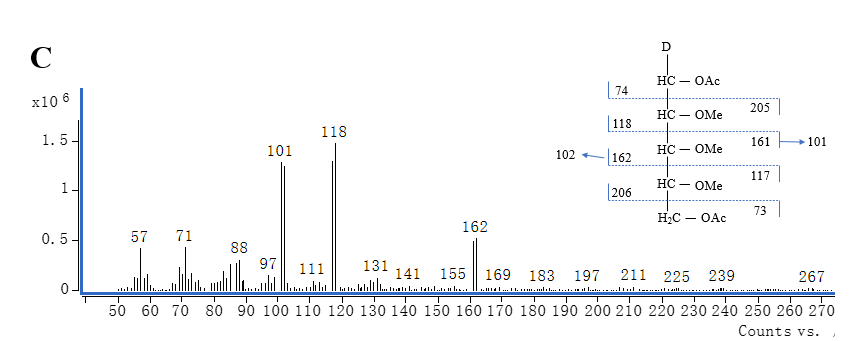


**Figure S3.** Mass spectra of peak at 8.643 min; PMAA: 1,5-di-*O*-acetyl-2,3,4-tri-*O*-methyl arabinitoltype of linkage: Ara*p*-(1→


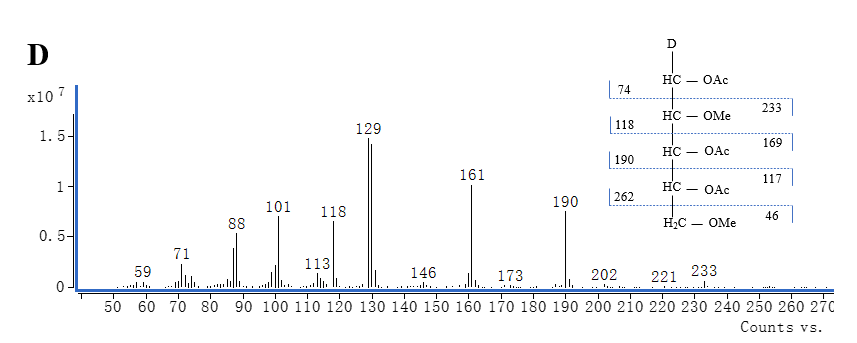


**Figure S3.** Mass spectra of peak 9.106 min; PMAA: 1,3,4-tri-*O*-acety1-2,5-di-*O*-methyl arabinitoltype of linkage: →3)-Ara*f*-(1→


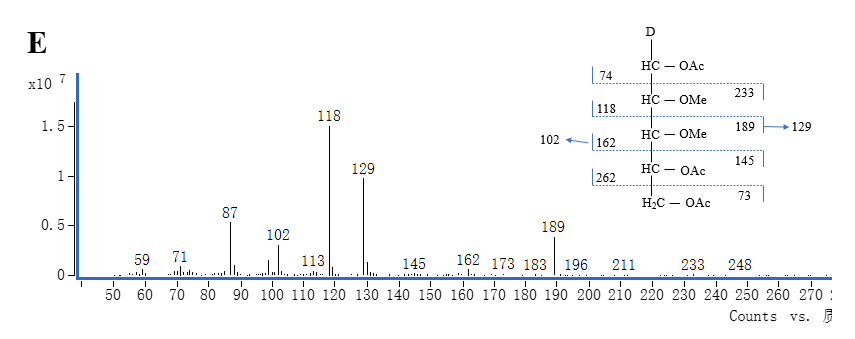


**Figure S3.** Mass spectra of peak at 9.398 min; PMAA: 1,4,5-tri-*O*-acety1-2,3-di-*O*-methyl arabinitoltype of linkage: →5)-Ara*f*-(1→


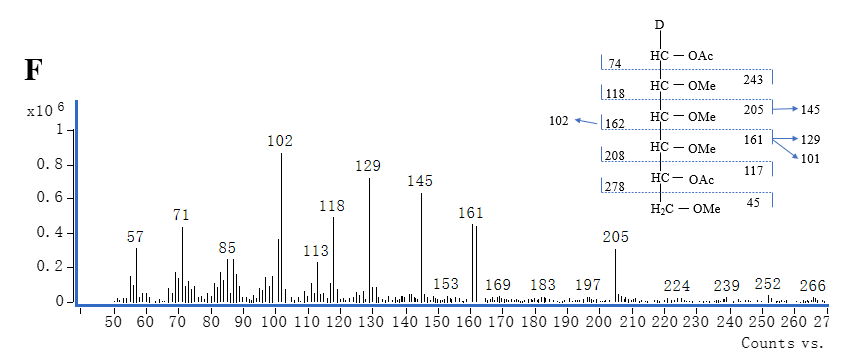


**Figure S3.** Mass spectra of peak at 9.830 min; PMAA: 1,5-tri-*O*-acetyi-2,3,4,6-tri-*O*-methyl gluctitoltype of linkage: Glc*p*-(1 →


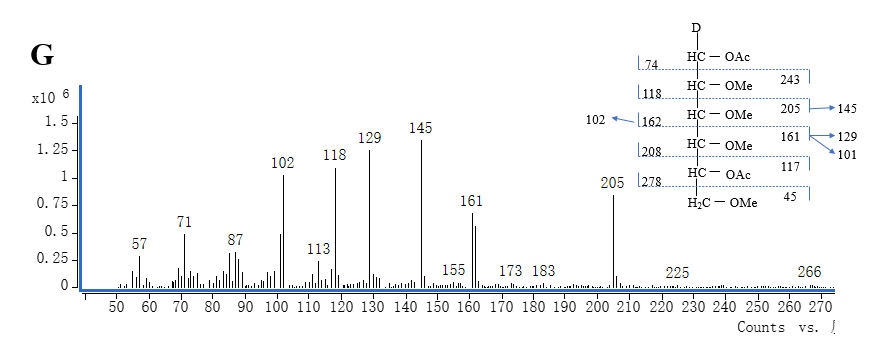


**Figure S3.** Mass spectra of peak at 10.047min; PMAA: 1,5-tri-*O*-acetyi-2,3,4,6-tri-*O*-methyl galactitoltype of linkage: Gal*p*-(1 →


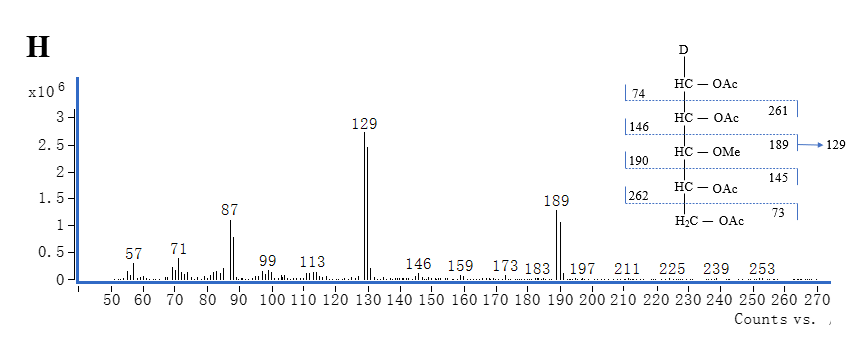


**Figure S3.** Mass spectra of peak at 10.356 min; PMAA: 1,2,4,5-tetra-*O*-acety1-3-di-*O*-methyl arabinitoltype of linkage: →2,5)-Ara*f*-(1 →


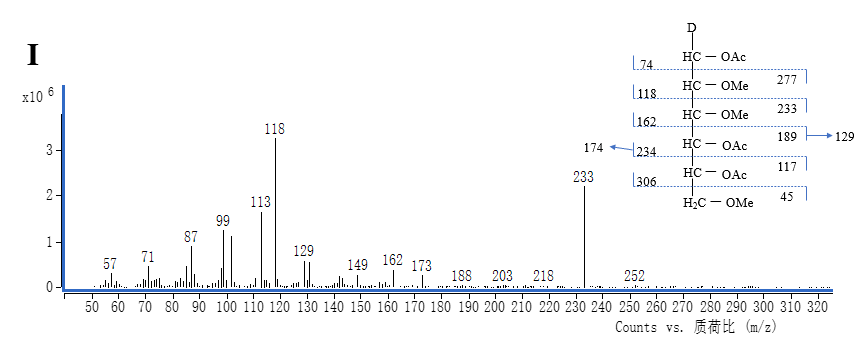


**Figure S3.** Mass spectra of peak at 10.973 min; PMAA: 1,4,5-tri-*O*-acety1-2,3,6-tri-*O*-methyl galactitoltype of linkage: →4)-Gal*p*-(1→


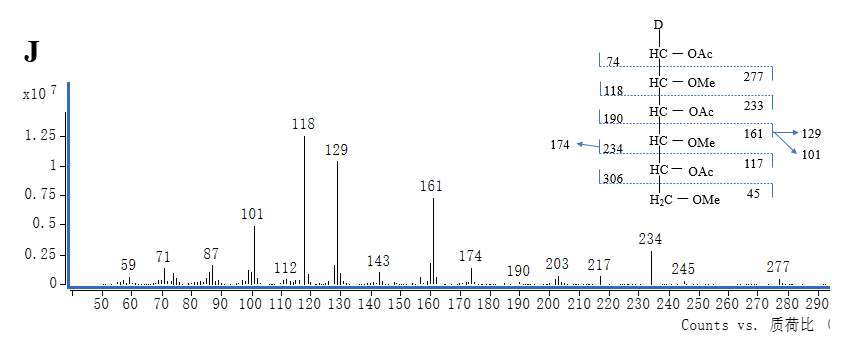


**Figure S3.** Mass spectra of peak at 11.083min; PMAA: 1,3,5-tri-*O*-acety1-2,4,6-tri-*O*-methyl galactitoltype of linkage: →3)-Gal*p*-(1→


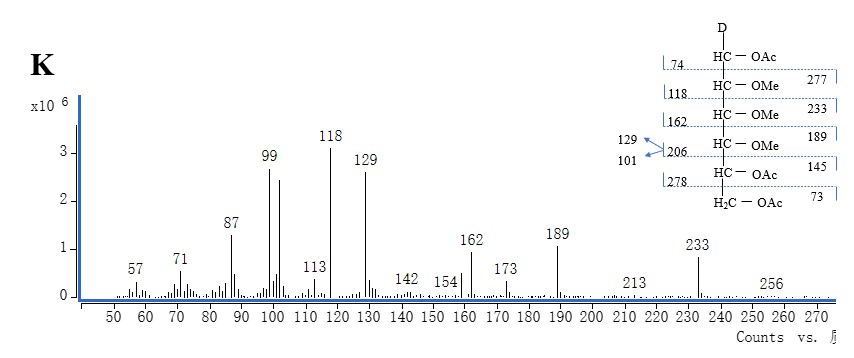


**Figure S3.** Mass spectra of peak at 11.583 min; PMAA: 1,5,6-tri-*O*-acety1-2,3,4-tri-*O*-methyl galactitoltype of linkage: →6)-Gal*p*-(1→


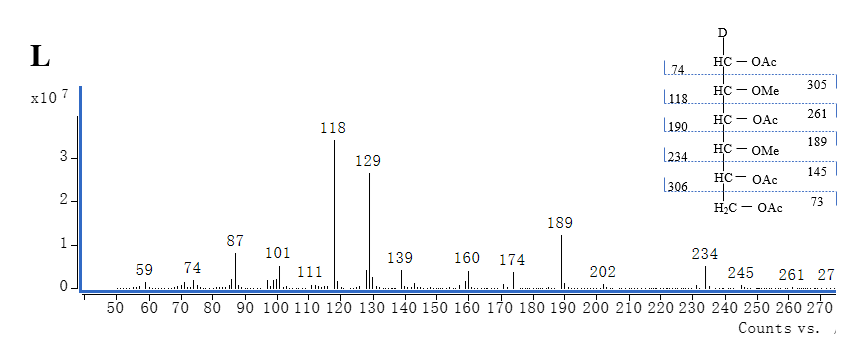


**Figure S3.** Mass spectra of peak at 12.809 min; PMAA: 1,3,5,6-tetra-*O*-acetyl-2,4-di-*O*-methyl galactitoltype of linkage: →3,6)-Gal*p*-(1→


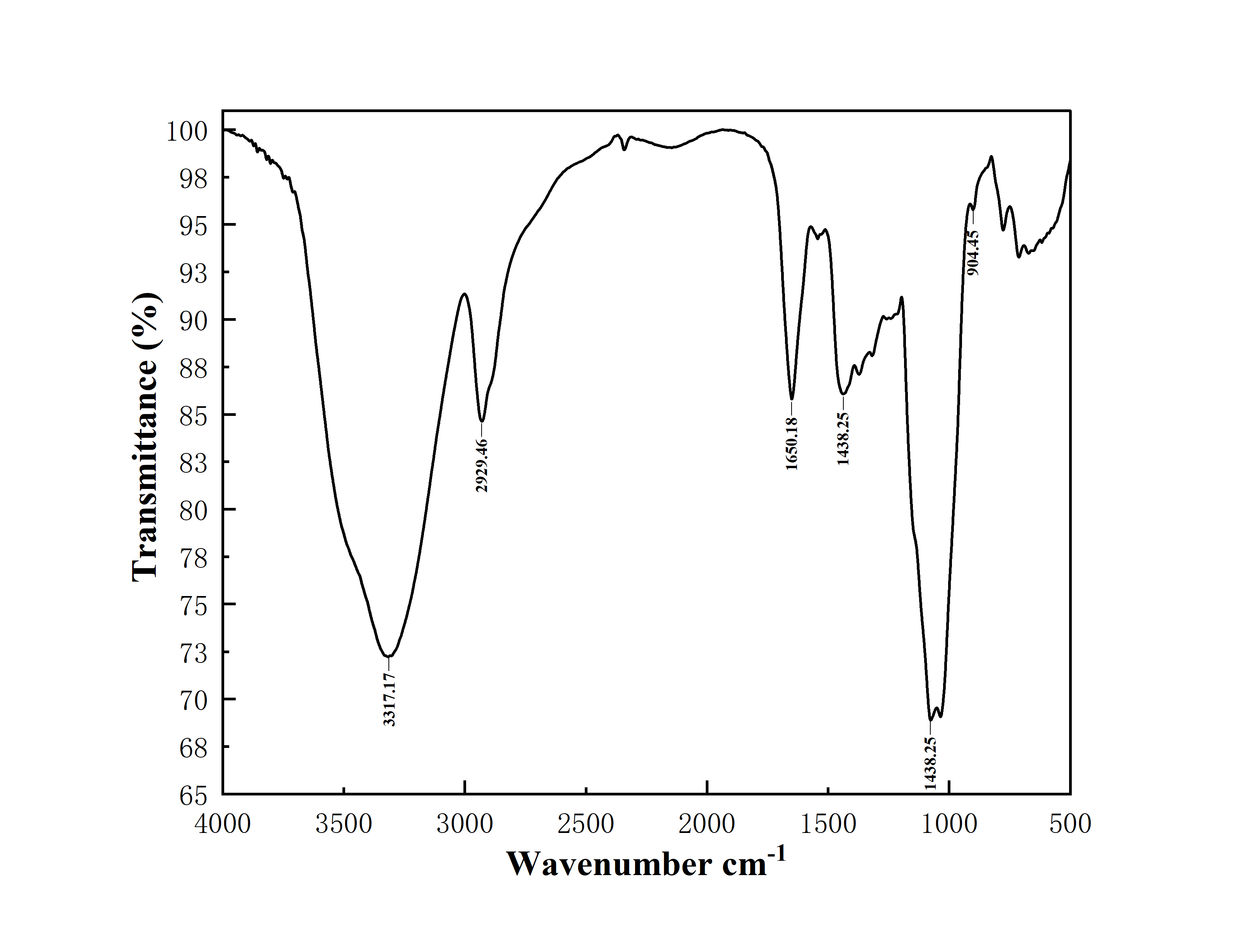


**Figure S4.** IR spectra of LFP-80-W1.


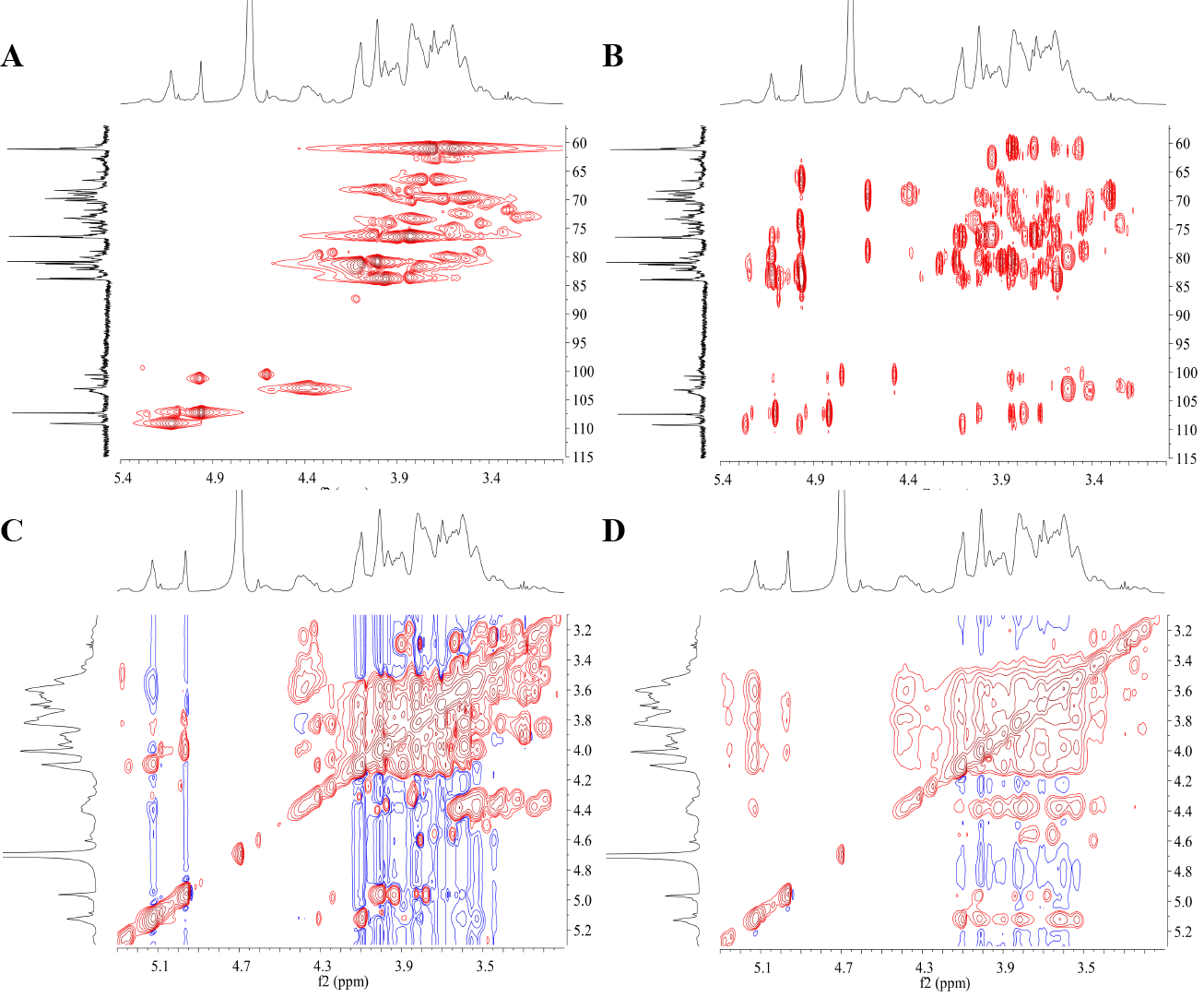


**Figure S5.** The full HSQC (A), HMBC (B), TOCSY (C) and NOESY (D) spectrum.
